# Operando Spatial Pressure Mapping Analysis for Prototype Lithium Metal Pouch Cells Under Practical Conditions

**DOI:** 10.1002/advs.202304979

**Published:** 2023-10-09

**Authors:** Kyobin Park, Myungjae Lee, Jongchan Song, A. Reum Ha, Seongmin Ha, Seunghyeon Jo, Juyeop Song, Seung Hyun Choi, Wonkeun Kim, Kyunghan Ryu, Jaewook Nam, Kyu Tae Lee

**Affiliations:** ^1^ School of Chemical and Biological Engineering Institute of Chemical Processes Seoul National University 1 Gwanak‐ro, Gwanak‐gu Seoul 08826 Republic of Korea; ^2^ Hyundai Motor Company 37 Cheoldobangmulgwan‐ro Uiwang‐si Gyeonggi‐do 16082 Republic of Korea

**Keywords:** electrochemical performance, failure mechanisms, inhomogeneity, lithium metal electrodes, operando, pouch cells, pressure

## Abstract

Monitoring and diagnosing the battery status in real‐time are of utmost importance for clarifying failure mechanism, improving battery performance, and ensuring safety, particularly under fast charging conditions. Recently, advanced operando techniques have been developed to observe changes in the microstructures of lithium deposits using laboratory‐scale cell designs, focusing on understanding the nature of Li metal electrodes. However, the macroscopic spatial inhomogeneity of lithium electroplating/stripping in the prototype pressurized pouch cells has not been measured in real‐time under practical conditions. Herein, a new noninvasive operando technique, spatial pressure mapping analysis, is introduced to macroscopically and quantitatively measure spatial pressure changes in a pressurized pouch cell during cycling. Moreover, dynamic spatial changes in the macroscopic morphology of the lithium metal electrode are theoretically visualized by combining operando pressure mapping data with mechanical analyses of cell components. Additionally, under fast charging conditions, the direct correlation between abrupt capacity fading and sudden increases in spatial pressure distribution inhomogeneity is demonstrated through comparative analysis of pouch cells under various external pressures, electrolyte species, and electrolyte weight to cell capacity (e/c) ratios. This operando technique provides insights for assessing the current battery status and understanding the complex origin of cell degradation behavior in pressurized pouch cells.

## Graphical abstract

Operando technique of spatial pressure mapping analysis is demonstrated to macroscopically and quantitatively measure pressure inhomogeneities and lithium metal morphology evolutions in pressurized Li | LiNi_0.8_Mn_0.1_Co_0.1_O_2_ (NMC811) pouch cells. The standard deviation of pressure distribution, obtained from the operando spatial pressure mapping analysis is considered a new indicator for determining the state‐of‐health of a battery, particularly under fast charging conditions.

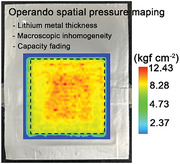

## 1 Introduction

Insistent demand for high‐energy‐density batteries has led to the development of Li metal anodes capable of outperforming the energy density limits of current Li‐ion batteries.^[^ ^1^ ^]^ Recent studies in the field of Li metal batteries (LMBs) have shown promises in the electrochemical performance of Li metal electrodes using innovative strategies, such as rational solvent molecule designs,^[^ ^2^ ^]^ functional electrolyte additives,^[^ ^3^ ^]^ and lithiophilic materials.^[^ ^4^ ^]^ Currently, these efforts are not limited to improving materials themselves academically, but have extended to examining them in large‐scale prototype Li‐metal batteries for industry. For this reason, Li metal electrodes are demanded to be evaluated under practical conditions, such as high current density and areal‐capacity, thin Li metal electrodes (≤ 50 µm), high‐loading cathode materials (≥ 3 mAh cm^−2^),^[^ ^5^ ^]^ and low electrolyte weight/cell capacity (e/c) ratio (≤ 5 g Ah^−1^).^[^ ^6^ ^]^ Furthermore, since the cell types, such as coin and pouch cells, affect the electrochemical performance of Li metal,^[^ ^7^ ^]^ Li metal electrodes are preferred to be examined using a pressurized pouch cell, which is close to prototype LMBs. In particular, external pressure is a prerequisite for pouch cells to suppress Li dendrites growth, leading to improving coulombic efficiency and capacity retention.^[^ ^8^ ^]^

Numerous in situ and operando non‐destructive characterization techniques have been developed to investigate the failure mechanism of Li metal by rationally capturing the transient states of highly reactive Li metal anodes during cycling.^[^ ^7^, ^9^ ^]^ For examples, microstructures of solid electrolyte interphase (SEI) and Li metal deposits were examined using in situ/operando tools, such as optical microscopy,^[^ ^10^ ^]^ transmission electron microscopy (TEM),^[^ ^11^ ^]^ Raman spectroscopy,^[^ ^12^ ^] 7^Li nuclear magnetic resonance spectroscopy (NMR),^[^ ^13^ ^]^ electron paramagnetic resonance (EPR),^[^ ^14^ ^]^ atomic force microscopy (AFM),^[^ ^15^ ^]^ and X‐ray synchrotron‐based techniques.^[^ ^16^ ^]^ Unfortunately, despite their advanced nature, these techniques have several limitations, including i) that most analyses were carried out using a laboratory‐scale designed cell without external pressure, rather than a pressurized pouch cell, ii) and that only localized areas of Li metal were observed microscopically. To complement previous microscopic analyses, a new operando technique should be developed to macroscopically observe dynamic changes in Li metal during cycling, using pouch cells that operate under practical conditions.

In this study, we introduced a new non‐invasive operando technique of spatial pressure mapping analysis to macroscopically and quantitatively measure changes in spatial pressure distribution of a pressurized pouch cell (5 × 5 cm^2^), in which the areal sensing resolution is 0.0171 cm^2^, during cycling under practical conditions. We demonstrated that the spatial pressure of the pouch cells was linearly proportional to the thickness of Li metal electrode under various conditions, such as electrolyte species, charge/discharge voltages, and cycle numbers. Macroscopic spatial changes in the thickness of the Li metal electrode during cycling were then theoretically visualized by combining operando pressure mapping data with mechanical analyses of the pouch cell components. In addition, we clarified a correlation between capacity fading and inhomogeneity in the spatial pressure of Li | LiNi_0.8_Mn_0.1_Co_0.1_O_2_ (NMC811) pouch cell during cycling. We performed a comparative analysis of the factors influencing the pressure uniformity of the pressurized Li | NMC811 pouch cell with a fluorinated ether‐based localized high‐concentration electrolyte (LHCE) in terms of fast‐charging and the e/c ratio. Remarkably, under fast charging conditions, the discharge capacity started to decrease rapidly at the point that the standard deviation of pressure distribution increased rapidly, whereas the mean pressure gradually increased regardless of abrupt capacity fading. This implies that capacity fading under fast‐charging conditions was triggered by macroscopically inhomogeneous Li plating/stripping, rather than microscopic Li dendrite formation. However, the fully fluoro‐sulfonyl electrolyte gave rise to macroscopically homogeneous Li plating/stripping even at a high 3 C rate, leading to stable cycling of Li | NMC811 pouch cells without abrupt capacity fading under practical conditions, such as a lean electrolyte (e/c = 5), thin Li metal electrode (20 µm), and high‐loading cathode (4 mAh cm^−2^).

## 2 Results and Discussion

### 2.1 Observing Inhomogeneous Plating and Stripping of Lithium Metal Through Operando Spatial Pressure Mapping Analysis

We constructed a pressure mapping cell kit, in which a 2D pressure sensor was inserted between a force‐distributing plate and a pouch cell, as shown in Figure S1 (Supporting Information). The pressure mapping cell was also equipped with a load cell for controlling the initial applied pressure and performing pressure calibration. The 2D pressure sensor has 2304 nodes for an area of 6.27 × 6.27 cm^2^, and thus, the areal sensing resolution is 0.0171 cm^2^. Pressure values were recorded at intervals of 90 s for 0.1 and 1 C‐rates and at intervals of 30 s for 3 and 5 C‐rates. A pressure value of the sensor was calibrated with pressure values of the load cell in the range of 2–8 bar. For the assembly of the double‐stacked pouch cells (5 × 5 cm^2^ in area), one double‐sided cathode electrode of LiNi_0.8_Mn_0.1_Co_0.1_O_2_ (NMC811) and two Li metal foil electrodes (20 µm in thickness) were used. The mass loading and density of NMC811 were 17.6 mg cm^−2^ and 3.5 g cm^−3^, respectively, which is equivalent to an areal capacity of 3.2 mAh cm^−2^. The negative electrode capacity to positive electrode capacity ratio (n/p ratio) was fixed at 1.25. 2.5 m lithium bis(fluorosulfonyl)imide (LiFSI) in 1,2‐dimethoxyethane (DME)/1,1,2,2‐tetrafluoroethyl‐1H,1H,5H‐octafluoropentyl ether (TFOFE) (8/2, v/v) with 0.3 wt.% LiPO_2_F_2_ and 1 m lithium hexafluorophosphate (LiPF_6_) in ethylene carbonate (EC)/dimethyl carbonate (DMC) (1/1, v/v) were used as the target electrolyte (denoted as LHCE) and control electrolyte (denoted as EC/DMC), respectively. The pouch cells were charged and discharged under lean electrolyte conditions, with an electrolyte mass per cell capacity (e/c ratio) of 5 g Ah^−1^, unless specified otherwise.

**Figure 1:**
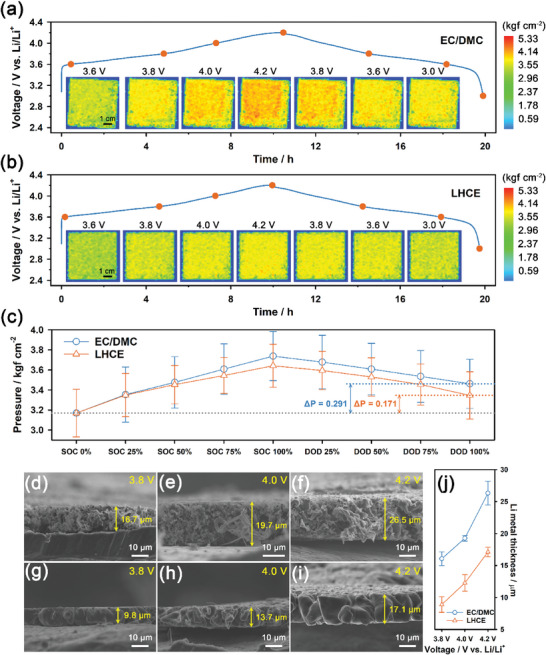
Voltage profiles and corresponding operando spatial pressure mapping images of the Li | NMC811 pouch cells with a) EC/DMC and b) LHCE electrolytes for various charge/discharge states at a 0.1 C rate and 3 bar, using an e/c ratio of 5 g Ah^−1^. c) Changes in the mean pressure and corresponding standard deviation of the Li | NMC811 pouch cells at various charge/discharge states. Pressure data were collected from 1260 activated pressure sensor nodes. Cross‐section SEM images of Li metal electrodes retrieved from the disassembled Li | NMC811 pouch cells with the d–f) EC/DMC electrolyte and g–i) LHCE electrolyte for various states of charge (3.8, 4.0, and 4.2 V) at a 0.1 C rate and 3 bar. j) Mean thickness of the Li metal electrode for various states of charge. Thickness data were collected from cross‐sectional SEM images of the Li metal electrode for five different positions obtained after electroplating.

**Figure** **1**a,b shows a comparison of operando pressure mapping images at different charge/discharge states and the corresponding voltage profiles of the Li | NMC811 cells at a 0.1 C rate and 3 bar for EC/DMC and LHCE electrolytes, respectively. A 1 C rate, which is equivalent to a specific current of 182 mA g^−1^ for NMC811, corresponds to a current density of 3.2 mA cm^−2^. The corresponding operando pressure mapping movies were presented in Movies S1 and S2 (Supporting Information). The pressure scale was color coded in the unit of kgf cm^−2^ from blue (0 kgf cm^−2^) to red (5.33 kgf cm^−2^). The unpressurized aluminum laminate film area on the edge side of the pouch cell was depicted in blue (0 kgf cm^−2^), while the pressurized electrode area of the pouch cell appears as a light green color (2.96 kgf cm^−2^). The pressure mapping images of the Li | NMC811 pouch cells for both EC/DMC and LHCE electrolytes gradually transitioned from a homogeneous light green to an inhomogeneous orange (4.14 kgf cm^−2^), as the cells were charged from 3.6 to 4.2 V. During discharge, the colors changed reversibly from orange to light green and yellow. This indicates that an increase and decrease in cell pressure during charge and discharge, respectively, are attributed to the plating and stripping of Li metal on the anode. Changes and inhomogeneity in the thickness of the NMC811 electrode during charge and discharge were negligible compared to the Li metal electrode because the volume change of the NMC811 cathode is known to be <6% during charge and discharge.^[^ ^17^ ^]^ In addition, the X‐ray diffraction (XRD) patterns of various selected areas in the NMC811 electrode retrieved from the pouch cell after charging to 4.1 V (vs Li/Li^+^) (Figure S2, Supporting Information) confirm the uniform state‐of‐charge (SOC) level of the NMC811 electrode across the entire area after charge.

Figure 1c shows the mean pressure values and corresponding standard deviations of the pressure data collected from 1260 sensor nodes at various SOC and depth‐of‐discharge (DOD) levels for EC/DMC and LHCE electrolytes. The standard deviation can be considered as an indicator that demonstrates the inhomogeneity of pressure distribution. It is notable that the changes and standard deviations in the thickness of the Li | NMC811 cell were significantly smaller for LHCE compared to EC/DMC. This implies that LHCE induced a more uniform and dense plating of Li metal compared to EC/DMC, which is coincident with the previous literatures.^[^ ^18^ ^]^ This is supported by cross‐sectional SEM images of the Li metal electrodes retrieved from the pouch cells at different SOC levels (Figure 1d–i). The Li | NMC811 pouch cells were disassembled after reaching various SOC levels, such as 3.8, 4.0, and 4.2 V (vs Li/Li^+^) at a 0.1 C rate and 3 bar. Figure 1j shows the mean thickness and corresponding standard deviation of the Li metal electrodes at various SOC levels, which was determined from five SEM images collected from five different locations for each sample. LHCE delivered an areal capacity of 3.42 mA h cm^−2^ during charging to 4.2 V, with the mean thickness of Li metal electroplated on the bare Li metal foil surface during charge being ≈17.1 µm. Note that bare Li metal foil (20 µm in thickness) delivered 4.01 ± 0.18 mA h cm^−2^ for full stripping (Figure S3, Supporting Information). This implies that the density of the electroplated Li metal was significantly high, which was nearly equal to that of the bare Li metal foil. In contrast, when EC/DMC delivered an areal capacity of 3.7 mA h cm^−2^ during charge to 4.2 V, the mean thickness of Li metal electroplated on the bare Li metal foil surface during charge was ≈26.5 µm. The density of electroplated Li metal for EC/DMC was significantly lower than that for LHCE. This is attributed to the fact that EC/DMC formed more porous and dendritic Li metal during electroplating compared to LHCE. Furthermore, the mean pressure of electroplated Li metal did not completely recover to its initial state after full stripping (discharging to 3.0 V vs Li/Li^+^), as shown in Figure 1c. This is due to the residual SEI layers formed on the Li metal surface during electroplating. In addition, the mean pressure of Li metal was higher in EC/DMC than in LHCE after full stripping. This implies that electrolyte decomposition was more severe in EC/DMC than in LHCE, resulting in a larger amount of residual SEI layers in EC/DMC, as shown in the SEM images of EC/DMC and LHCE after stripping (Figure S4, Supporting Information). We also compared the electrochemical performances of Li metal for EC/DMC and LHCE electrolytes using Li | Li symmetric and Li | Cu asymmetric cells (Figure S5, Supporting Information). LHCE exhibited significantly i) more stable capacity retention for Li | Li symmetric cells with an areal capacity of 1 mA h cm^−2^ at a current density of 1 mA cm^−2^ (Figure S5a, Supporting Information) and ii) higher coulombic efficiency for Li | Cu asymmetric cells with an areal capacity of 2 mA h cm^−2^ at a current density of 2 mA cm^−2^ (Figure S5b, Supporting Information) compared to EC/DMC. This was attributed to the formation of denser and less dendritic Li metal in LHCE compared to EC/DMC, as shown in the SEM images of Li metal retrieved from the Li | Cu asymmetric cells for EC/DMC and LHCE (Figure S5c,d, Supporting Information). This observation was also coincident with the cross‐sectional SEM images in Figure 1d–i.

**Figure 2:**
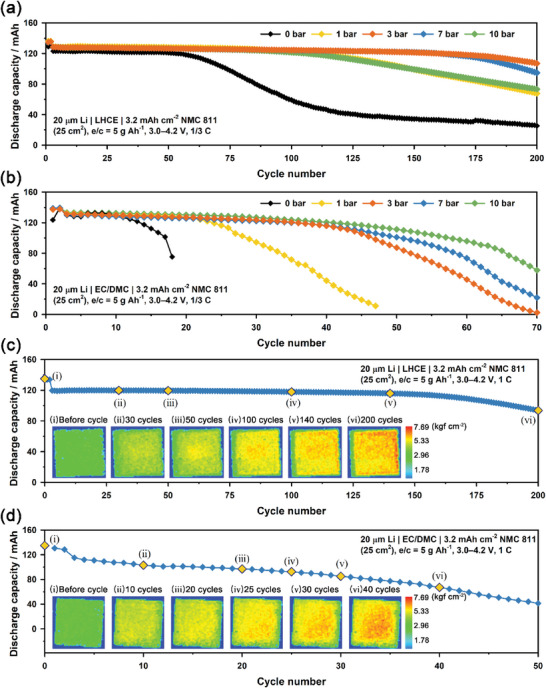
Cycle performance of the Li | NMC811 pouch cells with a) LHCE electrolyte and b) EC/DMC electrolyte at a C/3 rate under various pressure conditions, such as 0, 1, 3, 7, and 10 bar. Cycle performance and corresponding operando pressure mapping images of Li | NMC811 pouch cells with c) LHCE electrolyte and d) EC/DMC electrolyte for various cycle numbers at 4.2 V under conditions of a 1 C rate and 3 bar.

**Figure** **2**a compares the cycle performances of the Li | NMC811 pouch cells (5 × 5 cm^2^) containing LHCE (denoted as Li | LHCE | NMC811) at a C/3 rate for various cell pressure conditions, such as 0, 1, 3, 7, and 10 bar. The areal capacity of the NMC811 cathode was 3.2 mA h cm^−2^ and Li metal was 20 µm in thickness (corresponding to ≈4 mA h cm^−2^). The pressurized pouch cells showed stable capacity retention over 200 cycles, showing volcano‐type behavior as a function of the external pressure on the cell. The optimized cell pressure of pouch cells was in the range of 3–7 bar. The capacity retention of 3 bar was 83.3% after 200 cycles. In contrast, the cycle performance of the Li | NMC811 pouch cells containing EC/DMC (denoted as Li | EC/DMC | NMC811) was poor regardless of cell pressure conditions, although the external pressure slightly improved the capacity retention of Li | EC/DMC | NMC811 (Figure 2b). This is attributed to the fact that LiPF_6_ salt and carbonate solvent‐based electrolytes, such as EC/DMC, gave rise to severe chemical corrosion of Li metal.^[^ ^19^ ^]^ Figure 2c,d shows the cycle performances of Li | LHCE | NMC811, and Li | EC/DMC | NMC811 cells, respectively, and their corresponding operando pressure mapping images at a fully charged state (SOC = 100%) for various cycle numbers at a 1 C rate and 3 bar. The pressure mapping images of the Li | NMC811 pouch cells gradually and inhomogeneously changed from green to red. This reveals that Li metal was degraded unevenly during cycling even under high external pressure, regardless of the type of electrolyte. We found that the predominant failure mode of a large‐scale pouch cell was the macroscopic spatial inhomogeneity of electroplating and stripping, which will be discussed in a later section. Movies S3 and S4 (Supporting Information) show the dynamic inhomogeneous changes in the pressure distribution of Li | NMC811 pouch cells for EC/DMC and LHCE electrolytes, respectively, during cycling (from the 20th cycle).

### 2.2 Modeling the Macroscopic Spatial Changes of the Lithium Metal Electrode During Cycling

**Figure 3:**
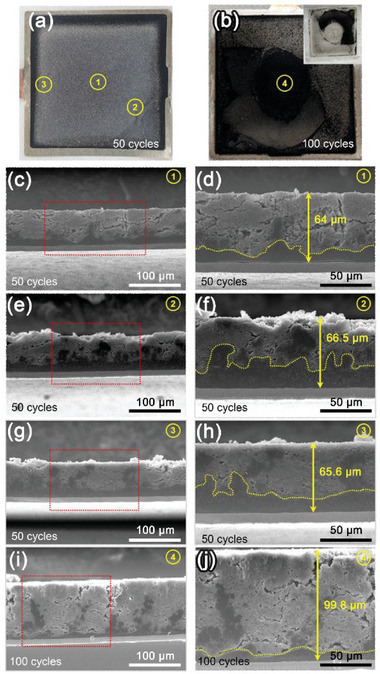
Photo images of Li metal electrodes retrieved from Li | LHCE | NMC811 pouch cells after a) 50 cycles and b) 100 cycles. The inset in (b) shows a photo image of the peeled electrode on the separator surface. Cryo‐cross‐sectional SEM images of Li metal electrodes retrieved from Li | LHCE | NMC811 pouch cells c–h) at 4.2 V after 50 cycles and i–j) after 100 cycles under conditions of 1 C rate and 3 bar. The magnified images of selected areas (indicated by red boxes) in (c), (e), (g), and (i) are presented as (d), (f), (h), and (j), respectively, for clarity.

We compared the cross‐sectional SEM images of Li metal electrodes retrieved after 50 and 100 cycles to demonstrate i) macroscopically inhomogeneous electroplating during cycling and ii) a correlation between spatial pressure and the thickness of Li metal electrodes. **Figure** **3**a,b shows the photo images of Li metal electrodes retrieved at 4.2 V (vs Li/Li^+^) after 50 and 100 cycles, respectively. The Li | LHCE | NMC811 pouch cells were cycled at a 1 C rate and 3 bar. In the case of 100 cycles, we observed that some areas of the Li metal electrode were peeled off. To obtain the cross‐sectional SEM images, we used a cryo‐cross‐section polisher (cryo‐CP) on the Li metal electrodes retrieved from a center region (1), an edge region (2), and a near‐tap region (3), after 50 cycles, as shown in Figure 3c–h, respectively. For clarity, Figure 3d,f, and h are magnified images of the selected areas (red boxes) in Figure 3c,e, and g, respectively. Regions (1–3) exhibited differences not only in the thickness of Li metal electrodes but also in the utilization of Li metal. The upper (porous) and lower (dense) regions, which are distinguished by yellow dotted lines, correspond to the utilized and non‐utilized areas of Li metal electrodes, respectively, during cycling. Figure 3i,j shows the cross‐sectional SEM images of the Li metal electrode retrieved from a center region (4) after 100 cycles. The thickness of the Li metal electrode, which was 20 µm before cycling, increased to ca. 65 and ca. 100 µm after 50 and 100 cycles, respectively. This supports that increases in the thickness of Li metal during cycling led to an increase in the pressure of Li | LHCE | NMC811 pouch cells. Eventually, this reveals that the inhomogeneous pressure distribution on the surface of Li | LHCE | NMC811 pouch cells was due to the uneven thickness of Li metal resulting from non‐uniform electroplating and stripping during cycling.

**Figure 4:**
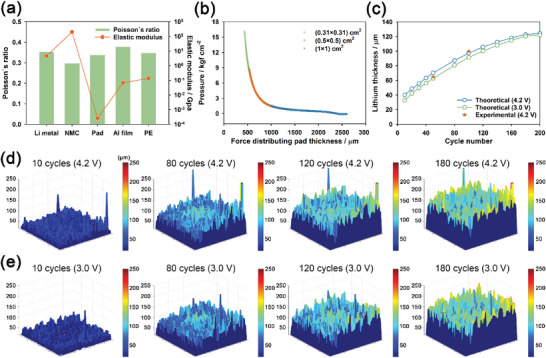
a) Elastic modulus and Poisson's ratio of the force‐distributing pad, aluminum laminate film, NMC cathode, PE separator, and Li metal used in a pressure pouch cell kit. b) The thickness of the force‐distributing pad as a function of the axial compression force applied on the force‐distributing pad. c) Theoretical calculation and experimental measurement of the Li metal thickness at fully charged (4.2 V) and discharged (3.0 V) states for various cycle numbers. Operando spatial morphology images of the Li metal electrode in the Li | LHCE | NMC811 pouch cell at d) 4.2 V and e) 3.0 V, respectively, during cycling under the same condition as in Figure 2c.

Moreover, we conducted theoretical calculations to demonstrate a correlation between the thickness of Li metal and the cell pressure in Li | LHCE | NMC811 pouch cells to provide direct support for the assertion that only the changes in Li metal thickness are responsible for altering the pressure of the Li | LHCE | NMC811 pouch cells. Considering all the layer components in Li | LHCE | NMC811 pouch cells, the total thickness can be expressed as follows:

(1) $$H_{\mathrm{Cell}}=\;H_{F}+\;H_{\mathrm{Al}}+\;H_{\mathrm{NMC}}+\;H_{\mathrm{PE}}+\;H_{\mathrm{Li}}$$

where *H*_Cell_, *H*_F_, *H*_Al_, *H*_NMC_, *H*_PE_, and *H*_Li_ refer to the total thickness of the cell and the individual thickness of the force‐distributing polyurethane pad, aluminum laminate film, NMC cathode, polyethylene (PE) separator, and Li metal electrode, respectively. Each layer's thickness depends on the cell pressure, whereas *H*_Cell_ remains constant during cycling due to mechanical constraint on the height imposed by our pressure mapping cell kit. The elastic modulus represents how the thickness of each layer changes with an increase in pressure. Consequently, the thickness of each layer can be expressed as a function of the elastic modulus, as follows:

(2) $$H_{\mathrm{Al}}\left(x,\;y,\;c\right)\;=\;H_{\mathrm{Al}}\left(x,\;y,\;0\right)\left(1-\;\frac{p\left(x,\;y,\;c\right)}{E_{\mathrm{Al}}}\right)$$

(3) $$H_{\mathrm{NMC}}\left(x,\;y,\;c\right)\;=\;H_{\mathrm{NMC}}\left(x,\;y,\;0\right)\left(1-\;\frac{p\left(x,\;y,\;c\right)}{E_{\mathrm{NMC}}}\right)$$

(4) $$H_{\mathrm{SE}}\left(x,\;y,\;c\right)\;=\;H_{\mathrm{SE}}\left(x,\;y,\;0\right)\left(1-\;\frac{p\left(x,y,c\right)}{E_{\mathrm{SE}}}\right)$$

(5) $$\begin{array}{ccc} H_{\mathrm{Li}}\;\left(x,\;y,\;c\right) & = & \left(H_{\mathrm{Li}}\left(x,\;y,\;0\right)\;+\;H_{\mathrm{Li},\;\mathrm{growth}}\left(x,\;y,\;c\right)\right)\\ & & \times \left(1-\;\frac{p\left(x,y,c\right)}{E_{\mathrm{Li}}}\right) \end{array}$$

(6) $$H_{F}\left(x,\;y,\;c\right)\;=\;H_{F}\left(x,\;y,\;0\right)\left(1-\;\frac{p\left(x,y,c\right)}{E_{F}}\right)$$

where *x*, *y*, *c*, *p*, and *E* are the horizontal and vertical position variables, cycle number, pressure, and elastic modulus, respectively. We measured the elastic modulus values of all layers from the normal strain–stress curve region, as shown in **Figure** **4**a and Figure S6 (Supporting Information). The elastic modulus of the force‐distributing pad was ≈10^3^ orders of magnitude lower than that of the other layers. This implies that the elastic deformation of the aluminum laminate film, NMC cathode, PE separator, and Li metal electrode was negligible compared to the force‐distributing pad. As a result, as the thickness of Li metal changes during cycling, only the thickness of the force‐distributing pad changes, whereas the thicknesses of the aluminum laminate film, NMC cathode, and PE separator remain almost unchanged. In addition, since *H*_Cell_ remains constant, *H*_Li,growth_ can be expressed as follows:

(7) $$H_{\mathrm{Li},\;\mathrm{growth}}\;\left(x,\;y,\;c\right)=H_{F}\;\left(x,\;y,\;0\right)-\;H_{F}\left(x,\;y,\;c\right)$$

However, in contrast to aluminum laminate film, NMC cathode, PE separator, and Li metal, the deformation of the force‐distributing pad was not linear with pressure within the pressure range of our experimental conditions. This implies that the elastic modulus of the force‐distributing pad cannot be considered as a constant value in Equation (6). For this reason, we measured the thickness of the force‐distributing pad as a function of the axial compression force applied to the force‐distributing pad using a DHR‐2 rheometer (TA instruments, U.S.A.) at 30 °C (Figure 4b). The same thickness‐force curves were obtained regardless of the size of the force‐distributing pad (0.0961, 0.25, and 1 cm^2^ in area), implying that the pad surface area remains almost unchanged although the pad thickness changes significantly with pressure. In addition, all the layers exhibited similar Poisson's ratio values in the range of 0.3–0.4, as shown in Figure 4a. This implies that the compression behavior of the force‐distributing pad is similar to that of the other layers.

Furthermore, we theoretically calculated the thickness of the Li metal electrode based on the mean pressure values of the pouch cells for various cycle numbers, using Equation (7), as shown in Figure 4c. The theoretical thickness of Li metal was then compared with the experimental thickness of Li metal that was estimated from the cross‐sectional SEM images for different cycle numbers in Figure 3. The theoretical thickness was almost coincident with the experimental thickness. This reveals that the height variation information of Li metal can be rationally obtained from the pressure variation of the cell. Moreover, the height variation distribution of two‐dimensional Li metal electrodes during cycling can be calculated from the operando pressure mapping values of Li | LHCE | NMC811 pouch cells, thereby enabling the visualization of changes in the macroscopic morphology of the Li metal electrode during cycling. Eventually, we obtained the operando morphology images of the Li metal electrode in the Li | LHCE | NMC811 pouch cell during cycling, as shown in Figure 4d,e. This demonstrates that Li metal grows inhomogeneously during cycling, leading to the failure of the Li metal pouch cell.

### 2.3 Correlation Between Macroscopic Pressure Inhomogeneity and Capacity Fading

**Figure 5:**
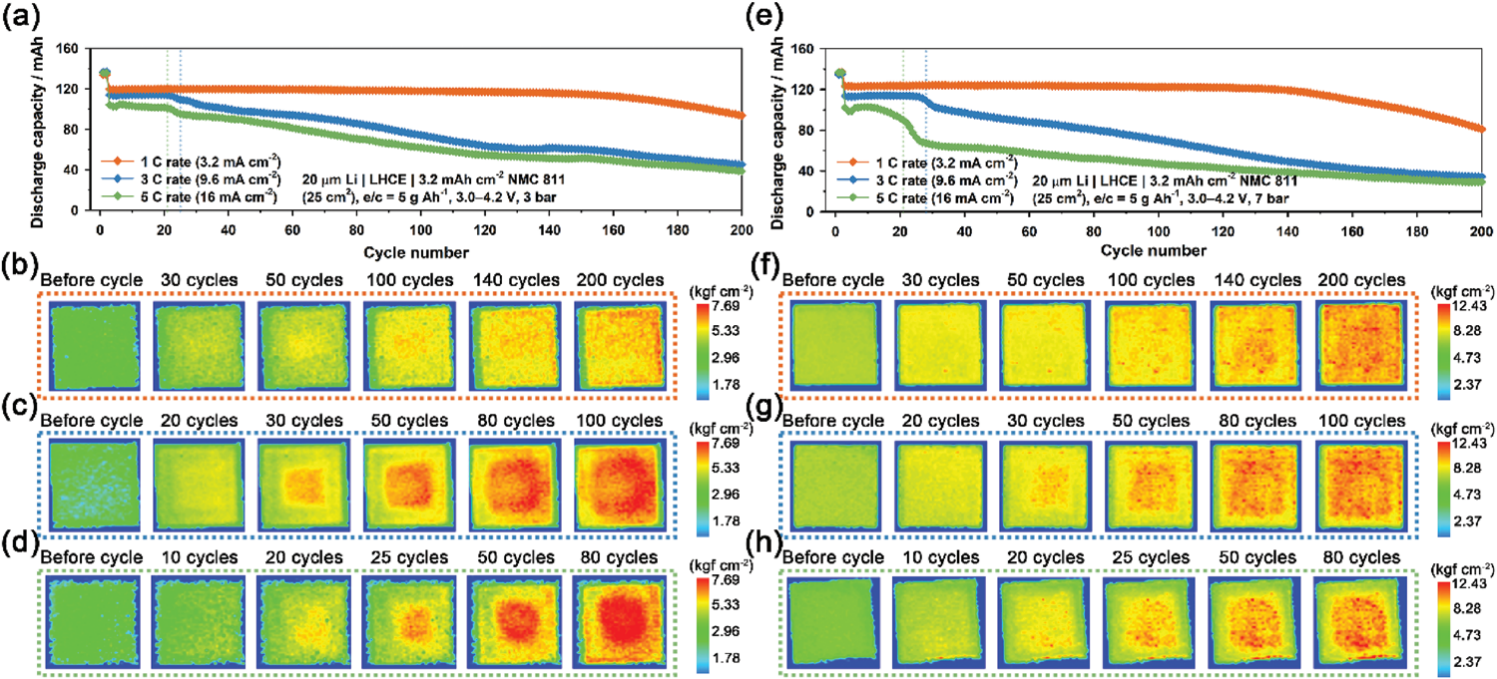
a) Cycle performance of the Li | LHCE | NMC811 pouch cells at 1 C, 3 C, and 5 C rate and 3 bar. Operando pressure mapping images of the corresponding Li | LHCE | NMC811 pouch cells at 4.2 V for various cycle numbers at b) 1 C, c) 3 C, and d) 5 C‐rates. e) Cycle performance of the Li | LHCE | NMC811 pouch cells at 1 C, 3 C, and 5 C rate and 7 bar. Operando pressure mapping images of the corresponding Li | LHCE | NMC811 pouch cells at 4.2 V for various cycle numbers at f) 1 C, g) 3 C, and h) 5 C‐rates.

To elucidate the correlation between spatial pressure inhomogeneity and capacity fading, we compared the operando pressure mapping images of Li | LHCE | NMC811 pouch cells at various C‐rates under two pressure conditions of 3 and 7 bar. **Figure** **5**a shows the cycle performance of Li | LHCE | NMC811 pouch cells at various C‐rates (1, 3, and 5 C) and 3 bar. As the C‐rate increased, the discharge capacity decreased, and the cycle performance gradually deteriorated. However, it is noteworthy that abrupt capacity fading was observed after ≈25 and 20 cycles at high C‐rates, such as 3 and 5 C‐rates, respectively. Figure 5b–d shows the operando pressure mapping images of Li | LHCE | NMC811 cells at 4.2 V at 1, 3, and 5 C‐rates, respectively, for different cycle numbers. Corresponding operando morphology images of the Li metal electrode at 3 C rate were also shown in Figure S7 (Supporting Information). We also compared the cycle performance of Li | LHCE | NMC811 pouch cells at various C‐rates under a higher‐pressure condition of 7 bar (Figure 5e). Similar to the case at 3 bar, the discharge capacity at 3 and 5 C‐rates abruptly decreased after ≈30 and 20 cycles, respectively. Figure 5f–h also presents the operando pressure mapping images of Li | LHCE | NMC811 cells at 4.2 V at 1, 3, and 5 C‐rates, respectively, for various cycle numbers. This implies that a sudden decline in the discharge capacity occurred under fast charging conditions, regardless of the applied external pressure value.

**Figure 6:**
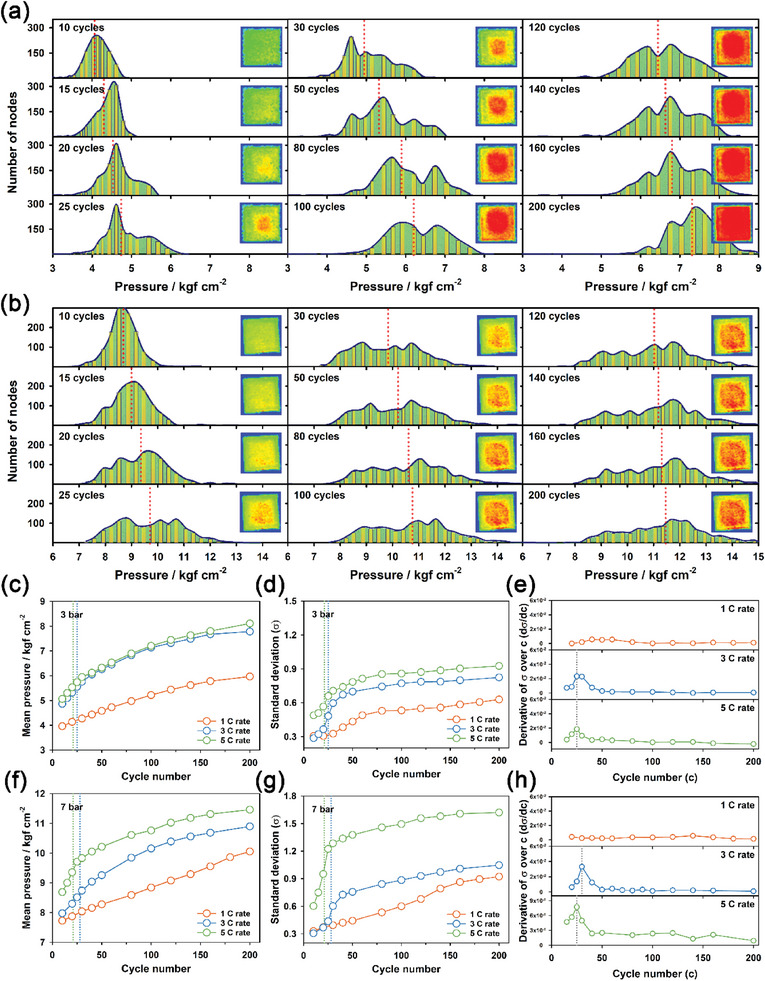
Pressure distribution profiles of activated sensor nodes in the pressurized Li | LHCE | NMC811 pouch cell for various cycle numbers at 5 C rate under the pressure conditions of a) 3 and b) 7 bar. c,f) Mean pressure, d,g) standard deviation, and e,h) derivative of standard deviation over cycle (d*σ*/dc) of the Li | LHCE | NMC811 pouch cells as a function of cycle number for various C‐rates under the pressure conditions of c–e) 3 and f–h) 7 bar, respectively.

Moreover, we quantitatively analyzed changes in the macroscopic spatial inhomogeneity of pressure distribution for the Li | LHCE | NMC811 pouch cells at fully charged (4.2 V vs Li/Li^+^) state during cycling under pressure conditions of 3–7 bar. **Figure** **6**a,b shows the pressure distribution profiles of the Li | LHCE | NMC811 pouch cells under 3 and 7 bar, respectively, for various cycle numbers at a 5 C rate. The pressure distribution profiles were reconstructed from spatial mapping images collected from 1260 activated sensor nodes, displaying the number of nodes as a function of pressure. Initially, the Li | LHCE | NMC811 pouch cells showed a narrow and monodisperse pressure distribution. However, as the cycle number increased, the pressure distribution of the pouch cells gradually changed to a more broadly distributed pattern. Similar pressure distribution profiles were also observed at fully charged and discharged states at a 1 C rate under pressure conditions of 3 and 7 bar (Figures S8–S10, Supporting Information). Figure 6c–e shows changes in the mean pressure value, corresponding standard deviation, and derivative of standard deviation over cycle, respectively, of Li | LHCE | NMC811 cells at 1, 3, and 5 C‐rates for various cycle numbers at 3 bar. Remarkably, changes in the standard deviation of pressure were found to be correlated with the capacity fading of the Li | LHCE | NMC811 cells, especially under fast charging conditions. The spatial inhomogeneity of pressure distribution can be described in terms of the standard deviation. The standard deviation of pressure distribution at 3 and 5 C‐rates abruptly increased after ≈25 and 20 cycles, respectively, at 3 bar (Figure 6e). We observed sudden changes in both the reversible capacity and the standard deviation of pressure at the almost identical cycle numbers for 3 and 5 C‐rates. The same behavior was also observed for the case at 7 bar. Figure 6f–h shows changes in the mean pressure value, corresponding standard deviation, and derivative of standard deviation over cycle, respectively, of Li | LHCE | NMC811 cells at 1, 3, and 5 C‐rates for various cycle numbers under a higher pressure condition of 7 bar. The cycle numbers at which the standard deviation of pressure distribution increased rapidly (≈30 cycles for 3 C‐rate and 20 cycles for 5 C‐rate) coincided with those at which the discharge capacity started to decrease rapidly. This reveals that an abrupt increase in spatial pressure inhomogeneity, driven by macroscopic nonuniform electroplating and stripping, gave rise to sudden capacity fading. Moreover, this indicates that the macroscopic inhomogeneous electroplating and stripping were accelerated to a greater extent with increasing current density.

We also examined the correlation between the inhomogeneity of pressure distribution and the abrupt capacity fading of the pressurized Li | LHCE | NMC811 pouch cell in terms of the e/c ratio. As the e/c ratio increased from 2.5 to 5 and 10 g Ah^−1^, the Li | LHCE | NMC811 pouch cell showed improved cycle retention of 84.6%, 87.7%, and 93.2% after 180 cycles at a 1 C rate and 3 bar, respectively (Figure S11, Supporting Information). The corresponding operando pressure mapping images at e/c = 10 for various cycle numbers were also presented in Figure S12 (Supporting Information). Moreover, we measured the mean cell pressure of the pressurized Li | LHCE | NMC811 pouch cell using the load cell (Figure S13, Supporting Information). The increase in mean cell pressure during cycling was more pronounced at e/c = 5 compared to e/c = 10. This indicates that the cycle stability of the pressurized Li | NMC811 pouch cell improved with a higher amount of electrolyte. Despite this fact, the sudden capacity fading was observed at the almost same cycle number regardless of the e/c ratio, as shown in Figure S14 (Supporting Information), although the absolute values of mean pressure and standard deviation were greater at e/c = 5 compared to e/c = 10. Remarkably, sudden changes in the standard deviation of pressure, which correspond to the inhomogeneity of pressure distribution, were directly correlated with the abrupt capacity fading of Li | LHCE | NMC811 cells (Figure S15, Supporting Information). This implies that the capacity retention of a prototype pouch cell under high current densities relies significantly on the macroscopic uniformity of electroplating and stripping.

### 2.4 Regulating Macroscopically Homogenous Lithium Plating and Stripping Under Fast Charging Conditions

**Figure 7:**
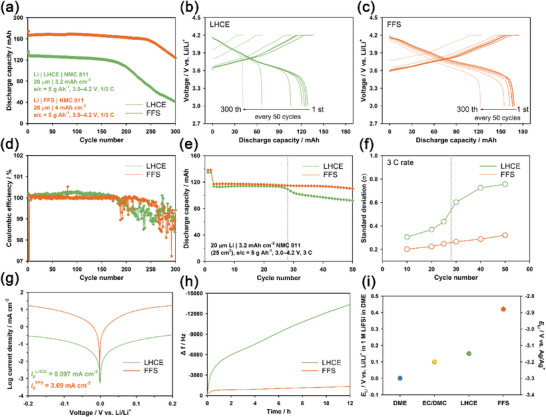
a) Cycle performance, b,c) voltage profiles, and d) coulombic efficiencies of the Li | NMC811 pouch cells for LHCE and FFS electrolytes at a C/3 rate and 3 bar. e) Cycle performance and f) standard deviation of pressure distribution for the Li | NMC811 pouch cells with LHCE and FFS electrolytes at a 3 C rate and 7 bar (e/c ratio = 5 g Ah^−1^). g) Tafel plots of the Li | Li symmetric cells for LHCE and FFS electrolytes. h) Changes in the resonant frequency of the Cu‐coated quartz electrodes as a function of time (EQCM) at 0 V (vs Li/Li^+^) for LHCE and FFS electrolytes. i) Redox potentials of Li metal electrodes in various electrolytes at 25 °C with reference to *E*_Li_ (*V* vs Ag/Ag^+^ and *V* vs 1 m LiFSI in DME).

Taking into account the aforementioned findings, the macroscopic inhomogeneity of Li metal plating and stripping behavior was notably exacerbated, particularly under fast charging conditions. This resulted in the failure of pressurized Li | NMC811 pouch cells. For this reason, regulating Li‐ion transport kinetics in the solid electrolyte interface (SEI) and electrolyte to facilitate homogeneous Li‐plating and stripping is key to improving electrochemical performance under fast charging conditions.^[^ ^20^ ^]^ Recently, fully fluoro‐sulfonyl electrolyte (FFS), introduced by Xue et al., showed significantly stable performance of LMBs.^[^ ^2^, ^21^ ^]^ Inspired by this study, we examined the FFS electrolyte for Li | NMC811 pouch cells to further substantiate the correlation between the macroscopic uniformity of electroplating and cycle performance. **Figure** **7**a shows the cycle performances of the Li | NMC811 pouch cells for the FFS and LHCE electrolytes at a C/3 rate and 3 bar. Although the areal capacity of the NMC811 cathode for the Li | FFS | NMC811 pouch cell (4 mA h cm^−2^) was even higher than that of Li | LHCE | NMC811 pouch cell (3.2 mA h cm^−2^), the pouch cell of Li | NMC811 for FFS electrolyte showed excellent capacity retention, such as 93.6% after 250 cycles, which was superior to that for LHCE electrolyte (53.2% after 250 cycles). Their corresponding voltage profiles and coulombic efficiencies were also presented in Figure 7b–d.

To demonstrate the role of macroscopically homogeneous Li‐plating and stripping in the improved capacity retention under fast charging conditions, we compared the cycle performances and standard deviations of pressure distribution for Li | NMC811 pouch cells using the FFS and LHCE electrolytes at a 3 C rate, as shown in Figure 7e,f, respectively. While sudden changes in both capacity fading and the standard deviation of pressure distribution were observed at the same cycle number for the Li | LHCE | NMC811 pouch cell, the Li | FFS | NMC811 pouch cell exhibited stable capacity retention with a slightly gradual change in the standard deviation of pressure distribution. This reveals that the FFS electrolyte promoted macroscopically uniform electroplating and stripping of the Li metal electrode, leading to improved capacity retention at high current density. The operando pressure mapping images of the Li | NMC811 pouch cells for the FFS and LHCE electrolytes at a 3 C rate were also presented in Figure S16 (Supporting Information).

To investigate the factors that induce macroscopically homogenous Li‐plating and stripping in the FFS electrolyte, we first compared the charge transfer kinetics of Li‐plating and stripping between the FFS and LHCE electrolytes. Figure 7g presents the Tafel plots of Li‐plating and stripping for the FFS and LHCE electrolytes, which were measured using Li | Li symmetric pouch cells. We measured the exchange current density (*i*_0_) from the Tafel plots. The exchange current density of the FFS electrolyte (3.69 mA cm^−2^) was ≈38 times larger than that of the LHCE electrolyte (0.097 mA cm^−2^), implying that the FFS electrolyte showed more facile charge transport kinetics compared to the LHCE.^[^ ^22^ ^]^ Second, we performed EQCM analysis to compare SEI layers derived from the FFS and LHCE electrolytes. Figure 7h compares changes in the resonant frequency of the working electrode as a function of time when 0 V (vs Li/Li^+^) was applied to the working electrode. A Cu‐coated quartz electrode and 100 µm Li metal electrode were used as working and counter electrodes, respectively. The Sauerbrey equation demonstrates that a change in resonant frequency of the working electrode is proportional to the mass variation of the working electrode.^[^ ^23^ ^]^ For this reason, a negative shift in the resonant frequency directly corresponds to an increase in the mass of Cu‐coated quartz electrode due to the formation of SEI layers at 0 V (vs Li/Li^+^). The resonant frequency change of the Cu‐coated quartz electrode in the FFS electrolyte was significantly smaller than that in the LHCE electrolyte, implying that the mass of the SEI layer derived from the FFS electrolyte was smaller than that from the LHCE electrolyte. This reveals that the FFS electrolyte was more reductively stable than the LHCE electrolyte.^[^ ^2a^ ^]^

To elucidate the origin of the improved reductive stability of the FFS electrolyte compared to the LHCE electrolyte, we compared the redox potentials of the Li metal electrode in various electrolytes, such as the FFS electrolyte, LHCE electrolyte, EC/DMC, and 1 m LiFSI in DME (denoted as DME), as shown in Figure 7i. The redox potentials of Li/Li^+^ were measured using the Ag/Ag^+^ reference electrode. The corresponding open circuit voltage (OCV) profiles of Li metal electrodes as a function of time are presented in Figure S17 (Supporting Information). The redox potential of Li/Li^+^ in FFS was significantly higher than those in LHCE, EC/DMC, and DME.^[^ ^24^ ^]^ The redox potential of Li/Li^+^ is dependent on the solvation structure of Li^+^ ions in electrolytes.^[^ ^24^ ^]^ The reductive stability of electrolytes is known to improve with increasing the redox potential of Li/Li^+^.^[^ ^24^ ^]^ For this reason, ether‐based electrolytes have demonstrated the improved reductive stability compared to carbonate‐based electrolytes. In particular, the redox potential of Li/Li^+^ in the FFS electrolyte was 0.27 V higher compared to that in the LHCE electrolyte. This implies that the reductive stability of the FFS electrolyte was improved compared to that of the LHCE electrolyte. As a result, the reductive decomposition of the FFS electrolyte on the Li metal surface was significantly suppressed, resulting in the formation of thin and stable SEI layers compared to other electrolytes. This suggests that the high exchange current density of the FFS electrolyte was attributable to the thin and stable SEI layers on the Li metal surface due to its improved reductive stability, which in turn leads to a higher degree of macroscopically uniform plating and stripping of Li metal during cycling.

## 3 Conclusion

We established a noninvasive operando technique of spatial pressure mapping analysis to demonstrate the failure mode of a pressurized Li | NMC811 pouch cell in terms of the evolution of macroscopic pressure inhomogeneities due to nonuniform electroplating and stripping of Li metal. Dynamic changes in the spatial pressure inhomogeneity were observed with respect to SOC level, electrolyte type, and cycle number. We also rationally visualized spatial changes in Li metal thickness during cycling by combining the theoretical calculation with the operando pressure distribution imaging analysis. In addition, we demonstrated that the standard deviation of pressure distribution, which is equivalent to the macroscopic spatial inhomogeneity of the Li metal thickness, obtained from the operando spatial pressure mapping analysis was considered a new indicator for determining the state‐of‐health (SoH) of a battery, particularly under fast charging conditions. The capacity fading of the Li | NMC811 pouch cells was directly correlated to the standard deviation of pressure distribution rather than the mean pressure of the pouch cell. In this regard, we examined the FFS electrolyte that showed the improved reductive stability compared to conventional electrolytes. The FFS electrolyte suppressed the macroscopically inhomogeneous electroplating and stripping of Li metal, leading to the excellent electrochemical performance, such as stable capacity retention of a double‐stacked pressurized Li | NMC811 pouch cell (25 cm^2^) over 250 cycles under practical conditions including a lean electrolyte (e/c ratio = 5 g Ah^−1^), a high‐loading of NMC811 (4.0 mAh cm^−2^), and thin Li metal electrode (20 µm in thickness, n/p = 1.0). Furthermore, this operando spatial pressure distribution mapping technique is not only limited to the demonstration of the failure mode of pressurized pouch cell but can be also applied to the battery management system (BMS) to ensure a reliable and safe operation.

## 4 Experimental Section

### Materials

LiPF_6_ (1 m) in EC/DMC (1/1, v/v) was purchased from Soulbrain, South Korea. LiFSI (2.5 m) in DME/TFOFE (8/2, v/v) + 0.3 wt.% LiPO_2_F_2_ and LiNi_0.8_Mn_0.1_Co_0.1_O_2_ (95.8 wt.% active material) cathode electrodes were provided by the Hyundai Motor group. Li metal foils (20 and 100 µm in thickness) were purchased from Honjo Metal, Japan.

### Materials Characterization

Cryo‐ion milling system was used for the preparation of cross‐sectioned samples (ArBlade 5000, HITACHI, Japan). A Field emission scanning electron microscope (FE‐SEM, JSM‐7800F Prime, JEOL, Japan) was used to observe the morphologies of Li metal. The XRD patterns of electrodes were acquired using a D2 Phaser with Cu K*α* radiation (*λ* = 1.5418 Å) operated in the 2*θ* range of 10–80°. The axial force was measured using DHR‐2 (TA instruments, New Castle, U.S.A.). Poisson's ratio and elastic modulus measurements were performed using Instron 3367 Universal testing Machine (Instron, U.S.A.).

### Electrochemical Measurements

The electrochemical performance of Li | LiNi_0.8_Mn_0.1_Co_0.1_O_2_ was examined in a voltage range of 3.0–4.2 V (vs Li/Li^+^). LiNi_0.8_Mn_0.1_Co_0.1_O_2_ was charged and discharged at various C‐rates (0.1, 1/3, 1, 3, and 5 C‐rates) using a constant current/constant voltage (CC/CV) mode where the cell voltage was held at 4.2 V (vs Li/Li^+^) until the current decayed to a 0.05 C rate. The mass loadings and density of LiNi_0.8_Mn_0.1_Co_0.1_O_2_ were 17.6 mg cm^−2^ and 3.5 g cm^−3^, respectively. Cycle performance was evaluated using a battery measurement system (WonATech WBCS 3000) at 30 °C under various pressures (0, 1, 3, 7, and 10 bar). The Tafel plots of Li | FFS | Li and Li | LHCE | Li cells were obtained at a scan rate of 1 mV s^−1^ using pouch cells. The redox couple of Ag/Ag^+^ (0.01 m AgNO_3_, 0.1 m tetrabutylammonium perchlorate (TBAP) in acetonitrile) was used as a reference electrode to measure the redox potential of Li metal in various electrolytes. The Ag/Ag^+^ reference electrode was calibrated with reference to the ferrocene/ferrocenium ion couple. The EQCM analyzer (Seiko EG&G, QCM922A) was used with a potentiostat (ZIVE). The Home‐made polyether ether ketone (PEEK) cell was used for EQCM analysis under Ar. Cu‐coated quartz electrode (0.6 µm surface roughness, 0.2 cm^2^) and 100 µm lithium metal electrode were used as working and counter electrodes, respectively.

### Fabrication of a Pressure Distribution Imaging Analysis Kit

SFM6000CX sensor (Kitronyx, South Korea) was used for pressure sensing with a maximum sensing area of 38.44 cm^2^, minimum to maximum pressure sensing range of 0.25–35 kgf cm^−2^, and sensing resolution of 0.0171 cm^2^ with 2304 nodes. The pressure distribution measurement system (Baikal‐II) with a maximum sensing speed of 30 Hz and Forcelab2 software v2.1.5 (Kitroxyx, South Korea) were used for pressure calibration and mapping analysis. A pressure sensor was placed between force‐distributing plate (SUS) and force‐distributing pad with a pouch cell. A BS‐205 load cell (Bongshin loadcell Co.) was attached under the force‐distributing SUS plate for calibration.

## Conflict of Interest

The authors declare no conflict of interest.

## Supporting information

Supporting Information

Supplemental Movie 1

Supplemental Movie 2

Supplemental Movie 3

Supplemental Movie 4

## Data Availability

The data that support the findings of this study are available from the corresponding author upon reasonable request.
