# Supplementary material for: Operando Spatial Pressure Mapping Analysis for Prototype Lithium Metal Pouch Cells Under Practical Conditions
Source: Adv Sci (Weinh). 2023 Oct 9;10(33):2304979. doi: 10.1002/advs.202304979 (PMC10667808; doi:10.1002/advs.202304979)
Supplement: Supplementary file 1 — Supporting Information [file ADVS-10-2304979-s001.pdf]

ADVANCED SCIENCE
Open Access

Supporting Information

for *Adv. Sci.*, DOI 10.1002/advs.202304979

Operando Spatial Pressure Mapping Analysis for Prototype Lithium Metal Pouch Cells Under Practical Conditions

Kyobin Park, Myungjae Lee, Jongchan Song, A. Reum Ha, Seongmin Ha, Seunghyeon Jo, Juyeop Song, Seung Hyun Choi, Wonkeun Kim, Kyunghan Ryu, Jaewook Nam and Kyu Tae Lee**

Supporting Information

Operando Spatial Pressure Mapping Analysis for Prototype Lithium Metal Pouch Cells under Practical Conditions

Kyobin Park[1], Myungjae Lee[1], Jongchan Song[2], A Reum Ha[2], Seongmin Ha[2], Seunghyeon Jo[1], Juyeop Song[1], Seunghyun Choi[1], Wonkeun Kim[2], Kyunghan Ryu[2], Jaewook Nam[1] and Kyu Tae Lee[1]**

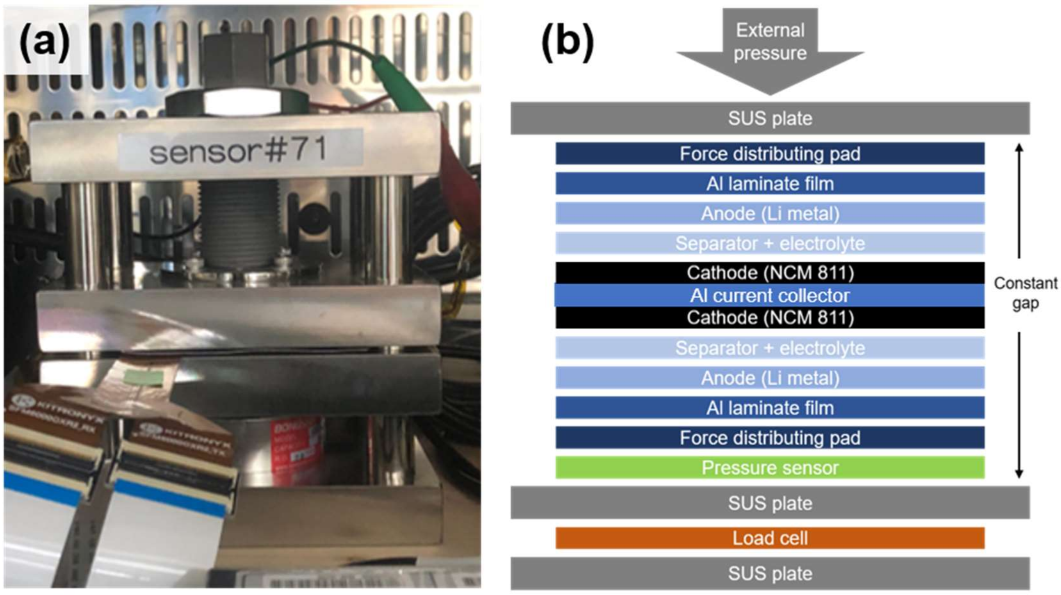

Figure S1. (a) Photograph and (b) schematic illustration of a pressure pouch cell kit with a pressure sensor and a load cell.

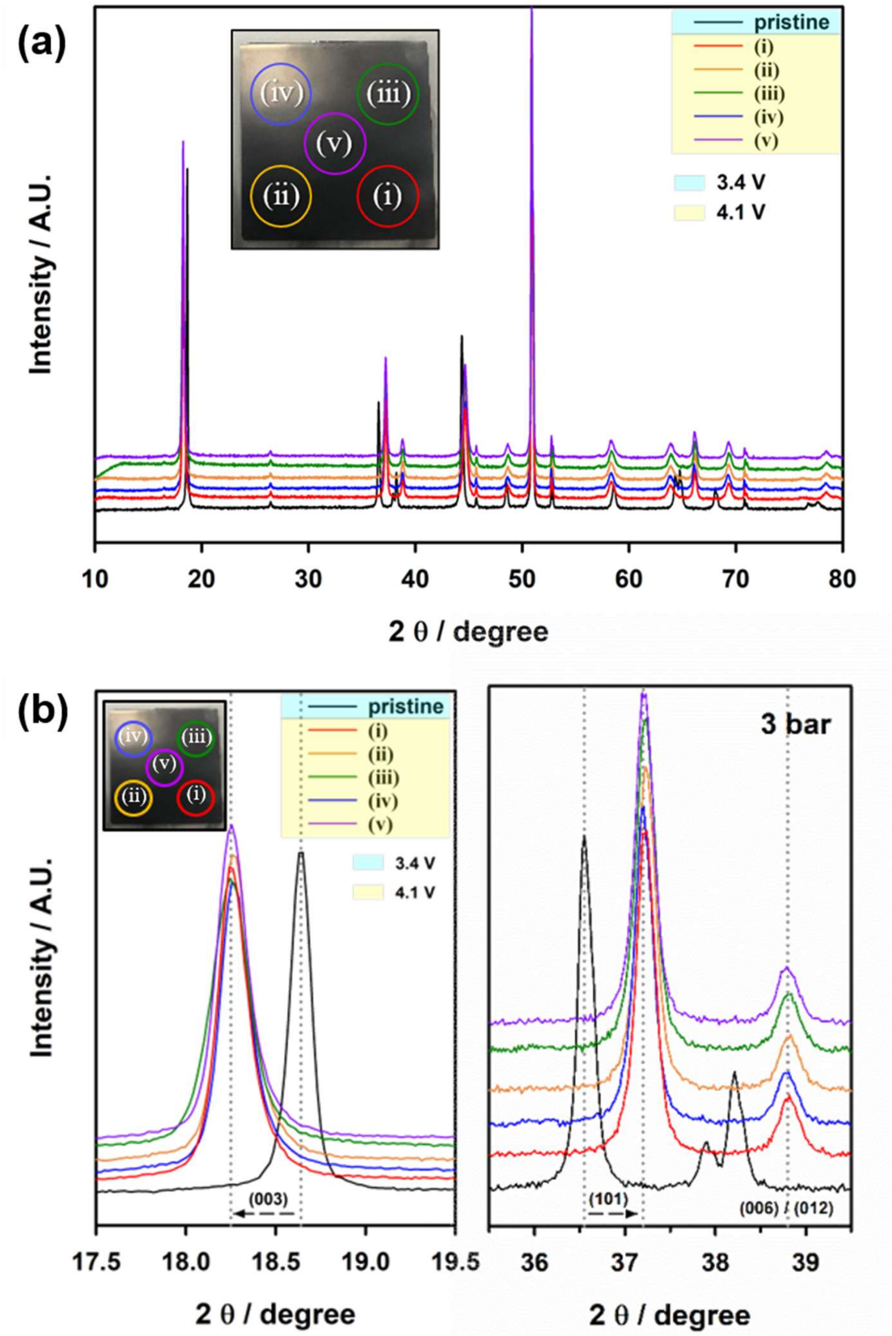

Figure S2. X-ray diffraction patterns of the various selected areas in the NMC811 electrode retrieved from the Li | NMC811 pouch cell after charging to 4.1 V (vs. Li/Li^+): (a) the entire 2θ range of 10-80° and (b) the selected ranges of 2θ.

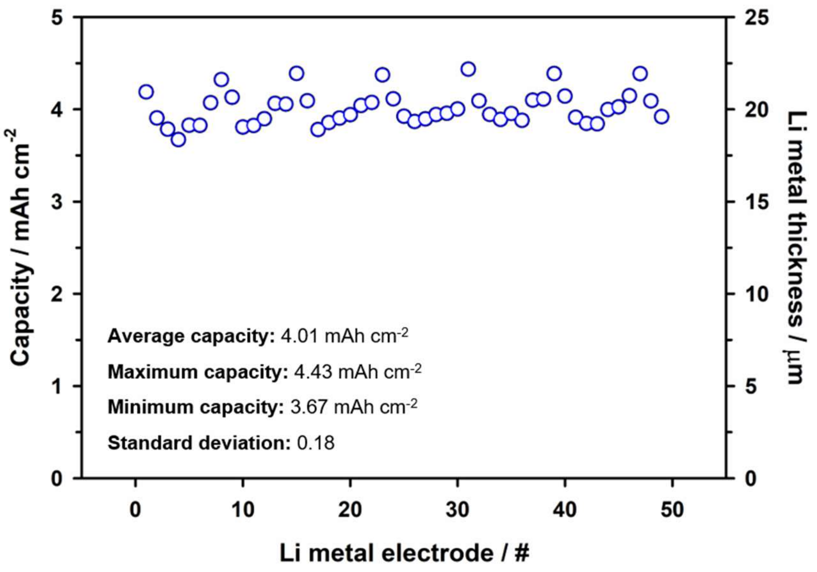

Figure S3. Stripping capacity and thickness of various selected areas (4 mm in diameter) in the Li metal electrode (20 μm in thickness and 25 cm^2 in area).

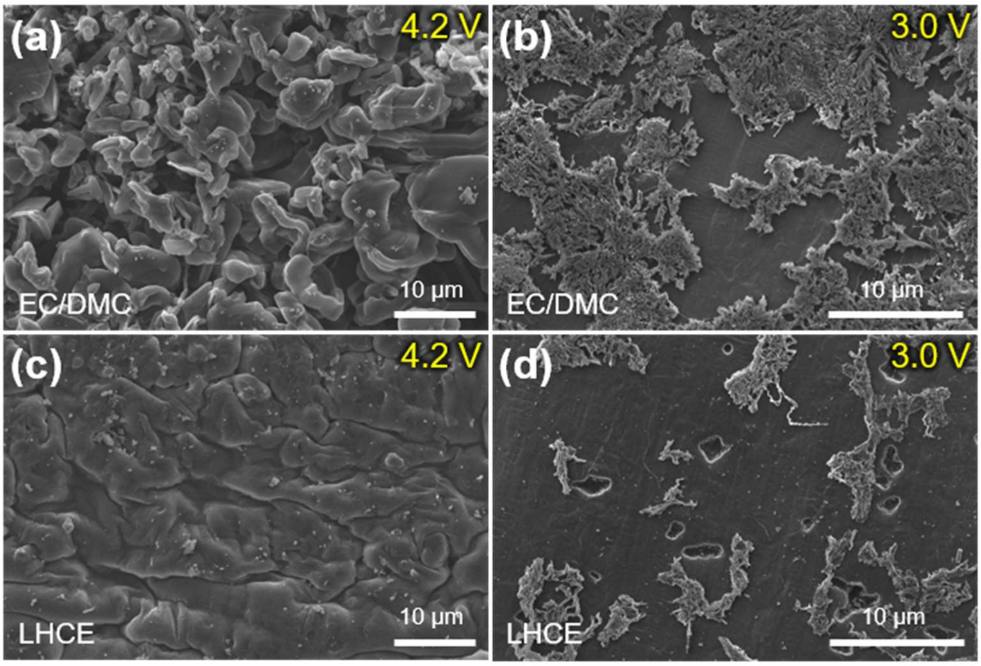

Figure S4. Top-view SEM images of Li metal electrodes retrieved from the Li | NMC811 pouch cells for (a, b) EC/DMC electrolyte and (c, d) LHCE electrolyte after initial charging to 4.2 V (vs. Li/Li^+) (a, c) and subsequent discharging to 3.0 V (vs. Li/Li^+) (b, d) at a 0.1 C rate and 3 bar.

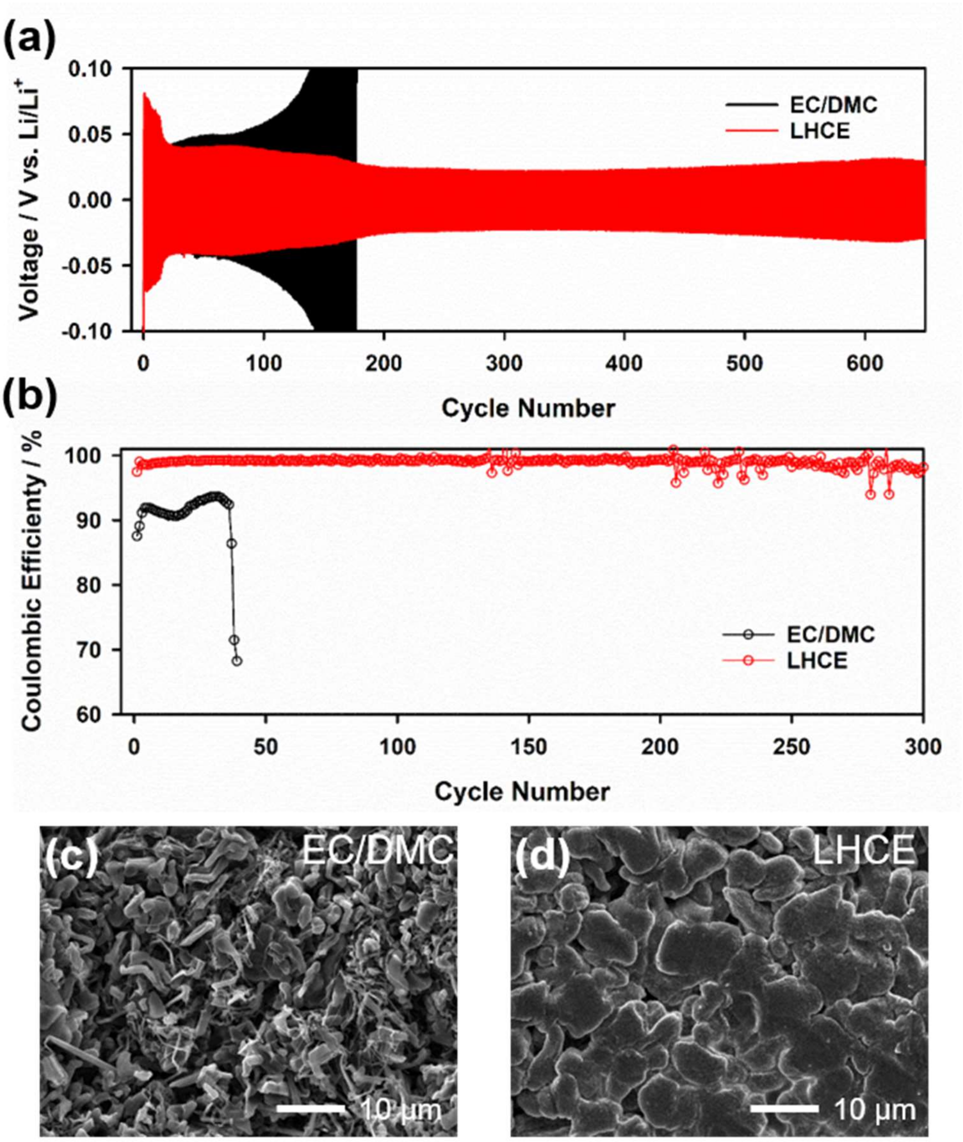

Figure S5. (a) Voltage profiles of Li | Li symmetric cells with an areal capacity of 1 mAh cm^{-2} at a current density of 1 mA cm^{-2}. (b) Coulombic efficiencies of Li | Cu cells at a current density of 2 mA cm^{-2}. 2 mAh cm^{-2} of Li was first plated on Cu electrode, followed by stripping until reaching 1 V (vs. Li/Li^{+}). SEM images of Li metal deposited on the Cu electrode with an areal capacity of 2 mAh cm^{-2} at a current density of 1 mA cm^{-2} for (c) EC/DMC and (d) LHCE electrolytes. These experiments were conducted using a 2032 coin-cell with Li metal (100 μm in thickness) and 40 μl of electrolyte.

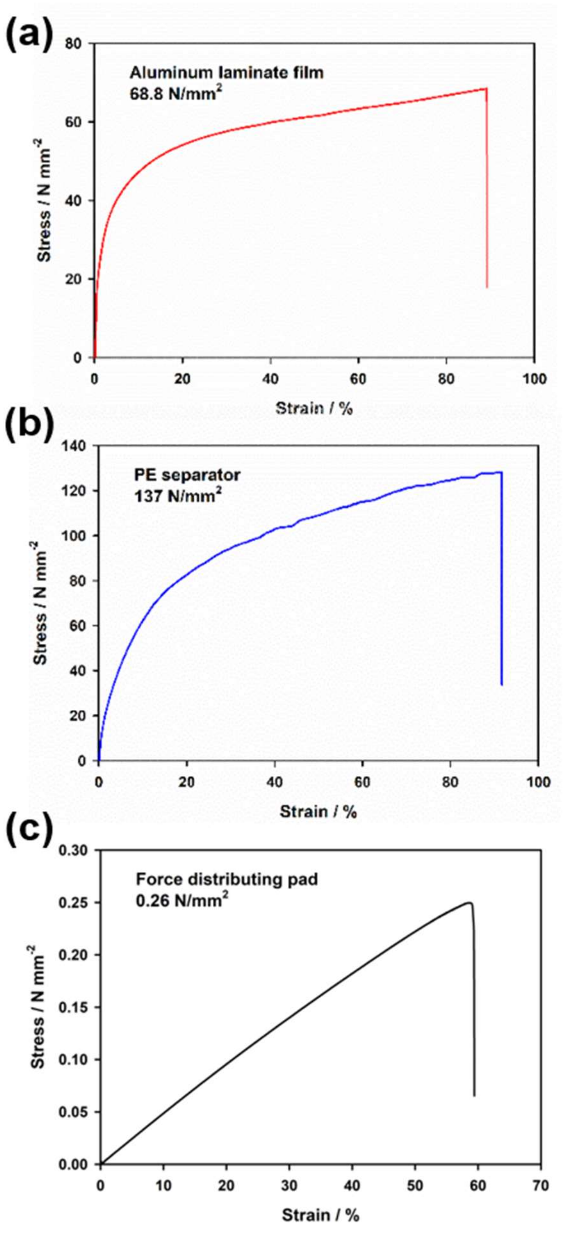

Figure S6. Normal strain-stress curves of (a) aluminum laminate film, (b) PE separator, and (c) force-distributing pad.

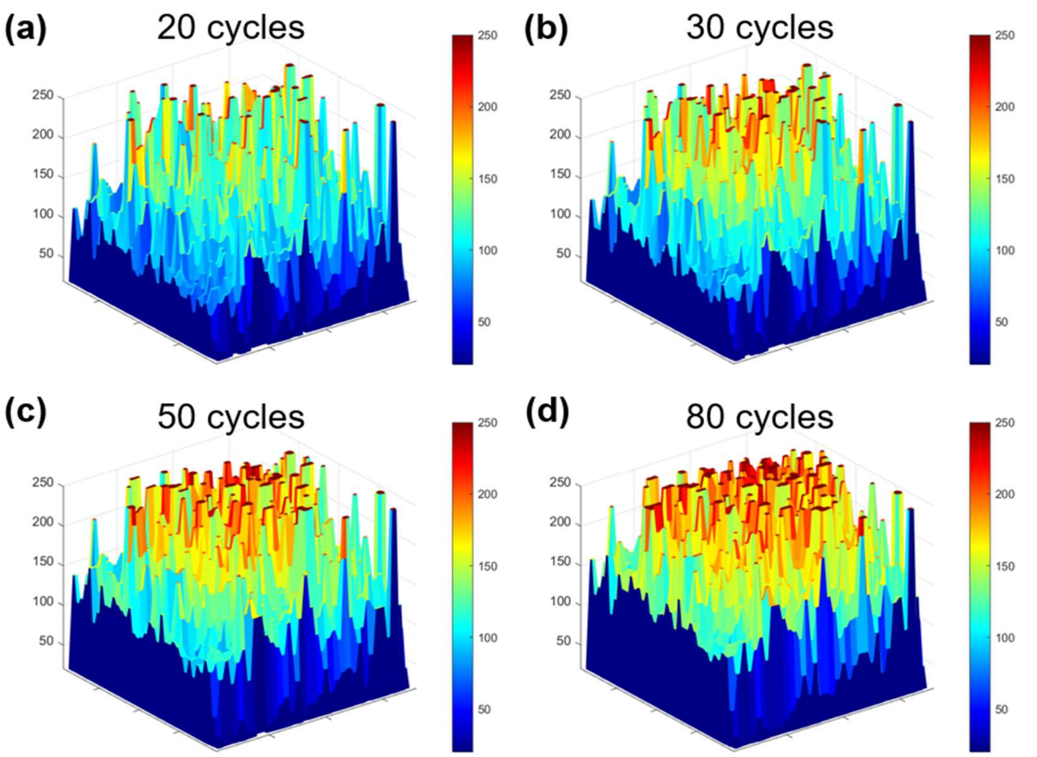

Figure S7. Operando spatial morphology images of the Li metal electrode in the Li | LHCE | NMC811 pouch cell at 4.2 V after (a) 20 cycles, (b) 30 cycles, (c) 50 cycles, and (d) 80 cycles, respectively, during cycling under the same condition as in Figure 5c.

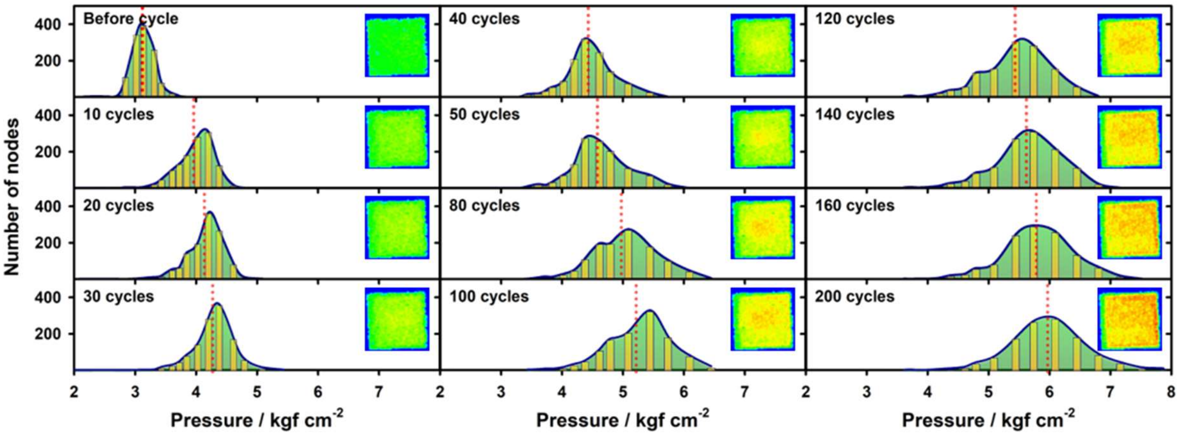

Figure S8. Pressure distribution profiles of activated sensor nodes in the pressurized Li | LHCE | NMC811 pouch cell at various cycle numbers at fully charged (4.2 V) state and 1 C rate under the pressure condition of 3 bar.

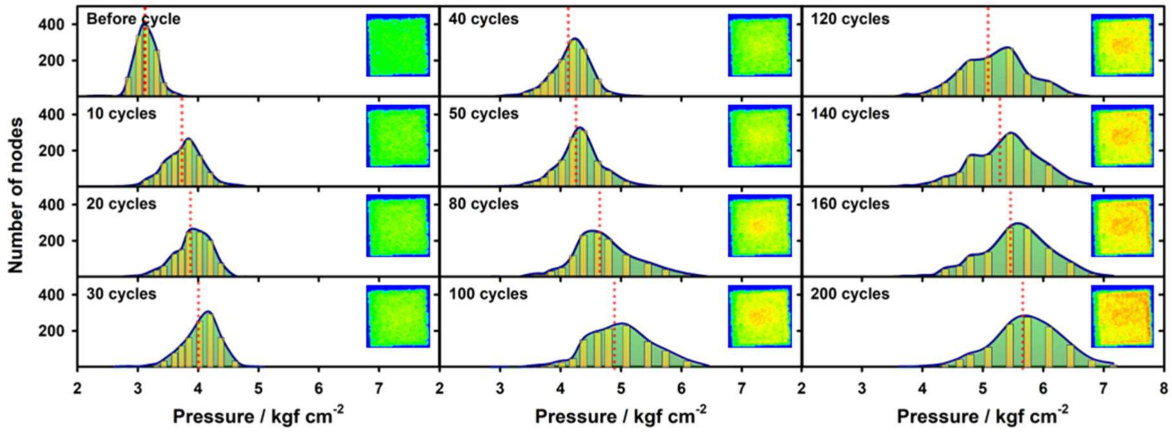

Figure S9. Pressure distribution profiles of activated sensor nodes in the pressurized Li | LHCE | NMC811 pouch cell at various cycle numbers at fully discharged (3.0 V) state and 1 C rate under the pressure condition of 3 bar.

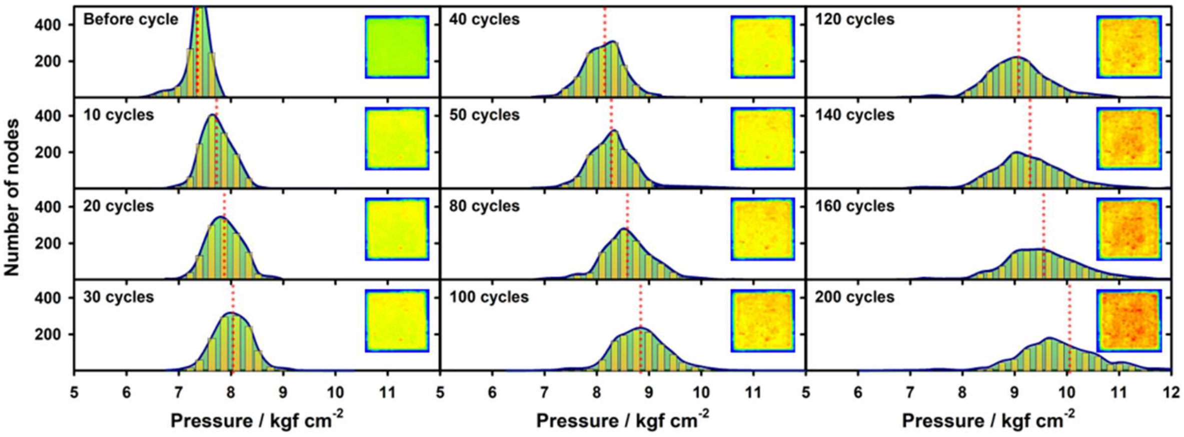

Figure S10. Pressure distribution profiles of activated sensor nodes in the pressurized Li | LHCE | NMC811 pouch cell at various cycle numbers at fully charged (4.2 V) state and 1 C rate under the pressure condition of 7 bar.

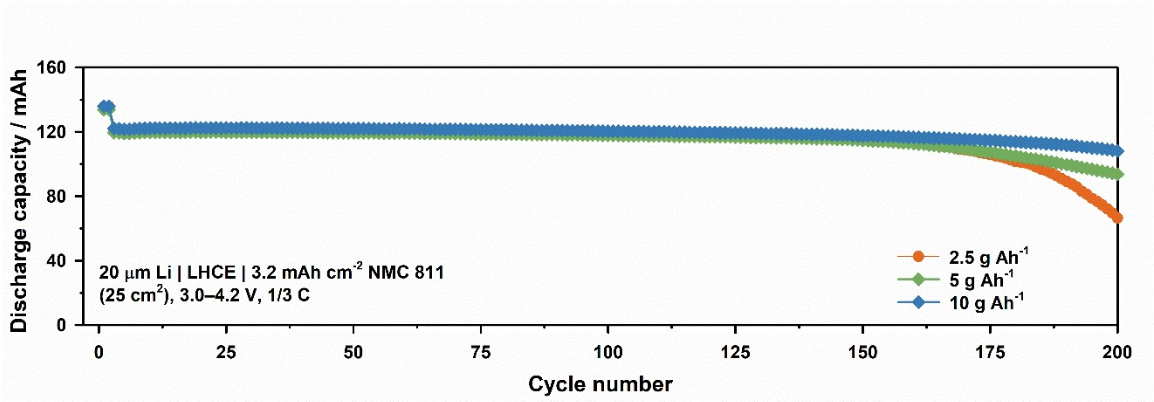

Figure S11. Cycle performance of the Li | LHCE | NMC811 pouch cells at 1 C rate and 3 bar with various e/c ratios (e/c=2.5, 5, and 10).

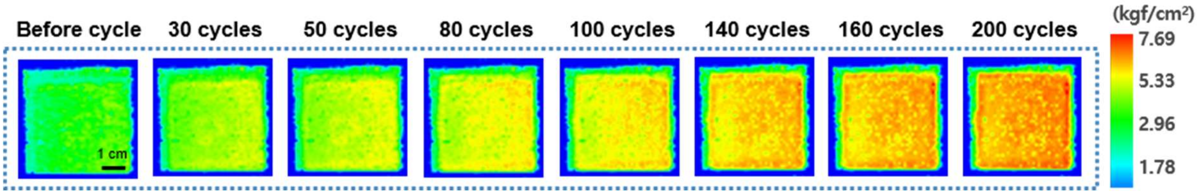

Figure S12. Operando pressure mapping images of the Li | LHCE | NMC811 pouch cell for various cycle numbers at 4.2 V under conditions of a 1 C rate, 3 bar and e/c=10.

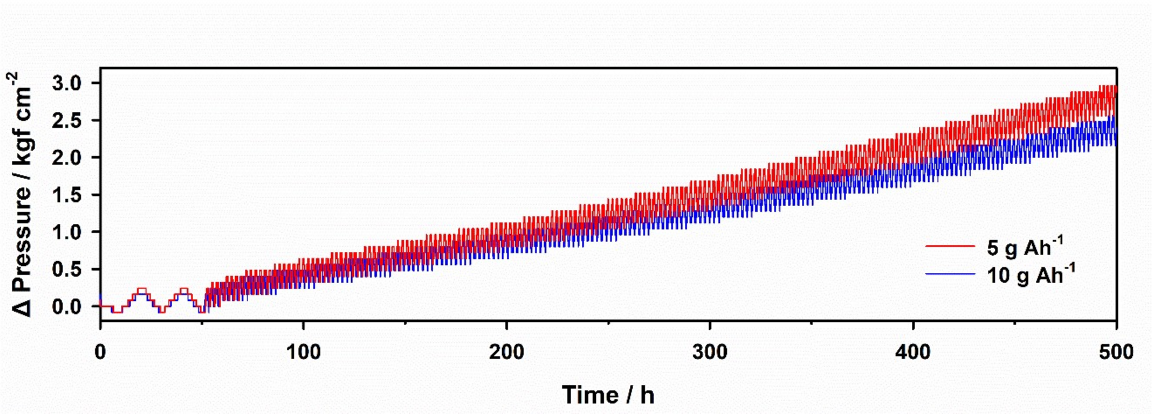

Figure S13. Changes in the cell pressure of Li | LHCE | NMC811 pouch cells during cycling measured using a load cell with two different e/c ratios of 5 and 10 g Ah^{-1} at a 1 C rate and 3 bar.

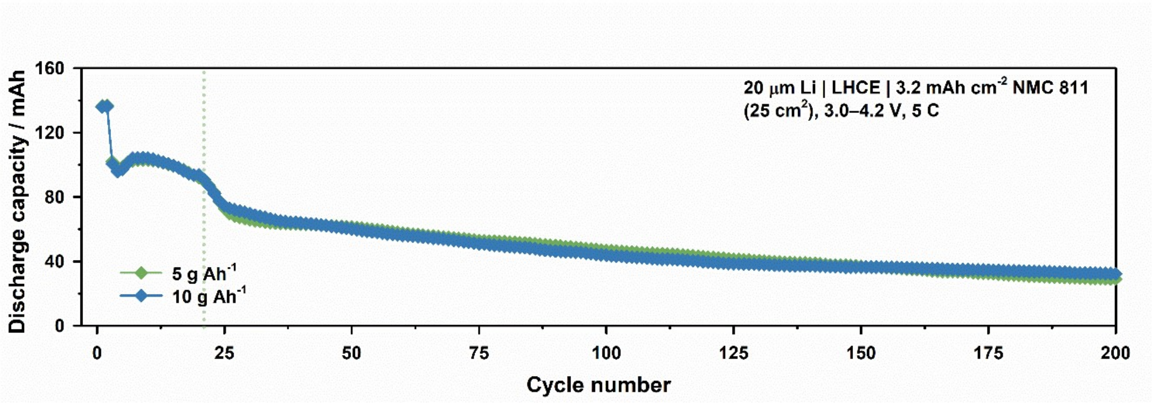

Figure S14. Cycle performance of the Li | LHCE | NMC811 pouch cells at 5 C rate and 7 bar for two different e/c ratios of 5 and 10 g Ah^{-1}.

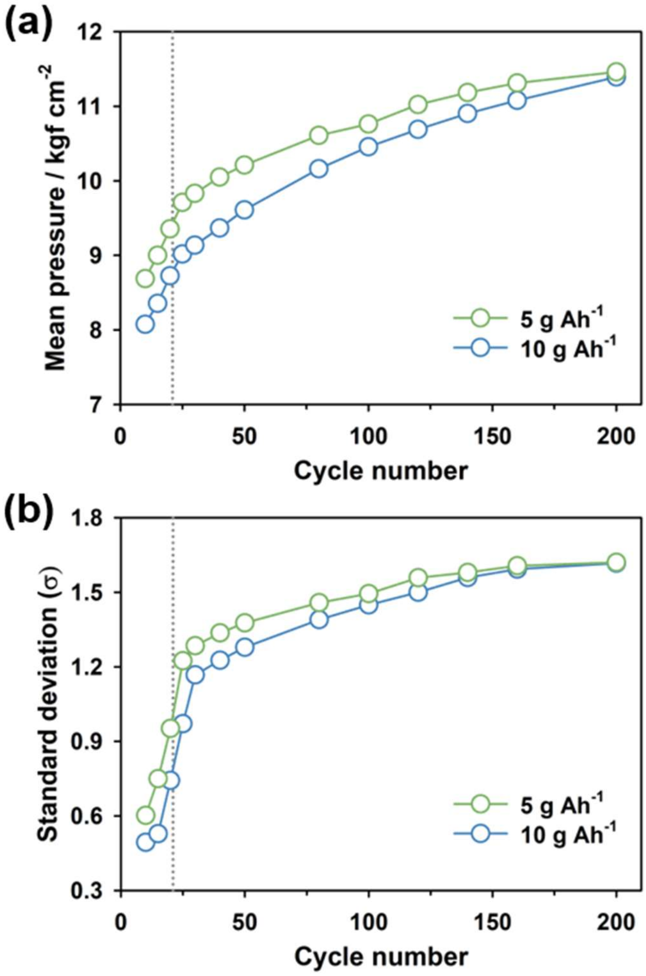

Figure S15. Changes in the (a) mean pressure and (b) standard deviation of the Li | LHCE | NMC811 pouch cells at a 5 C rate and 7 bar during cycling for two different e/c ratios of 5 and 10 g Ah^{-1}.

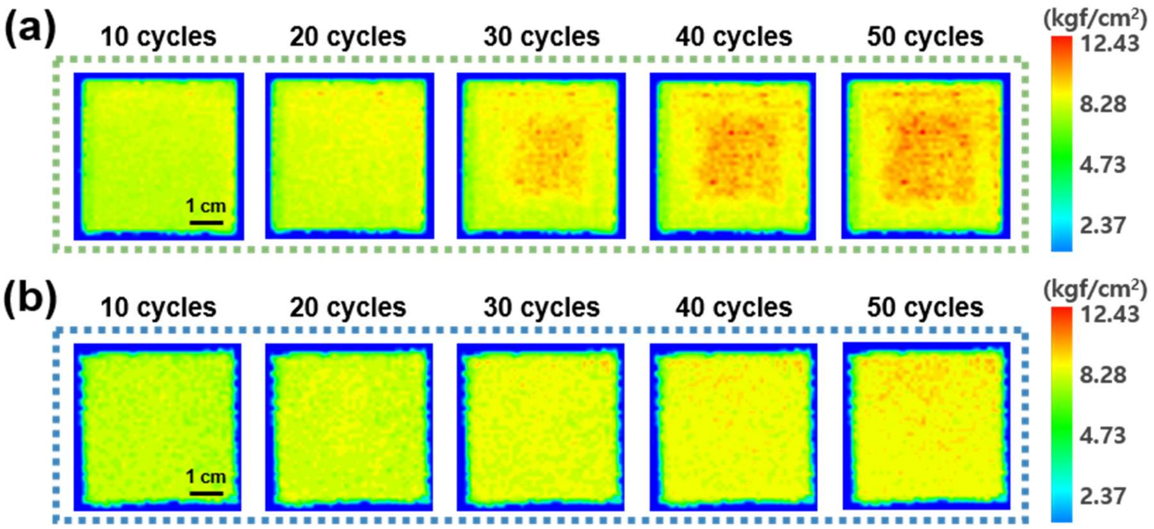

Figure S16. Operando pressure mapping images of the Li | NMC811 pouch cells with (a) LHCE and (b) FFS electrolytes at 4.2 V for various cycle numbers. The cells were cycled at a 3 C rate.

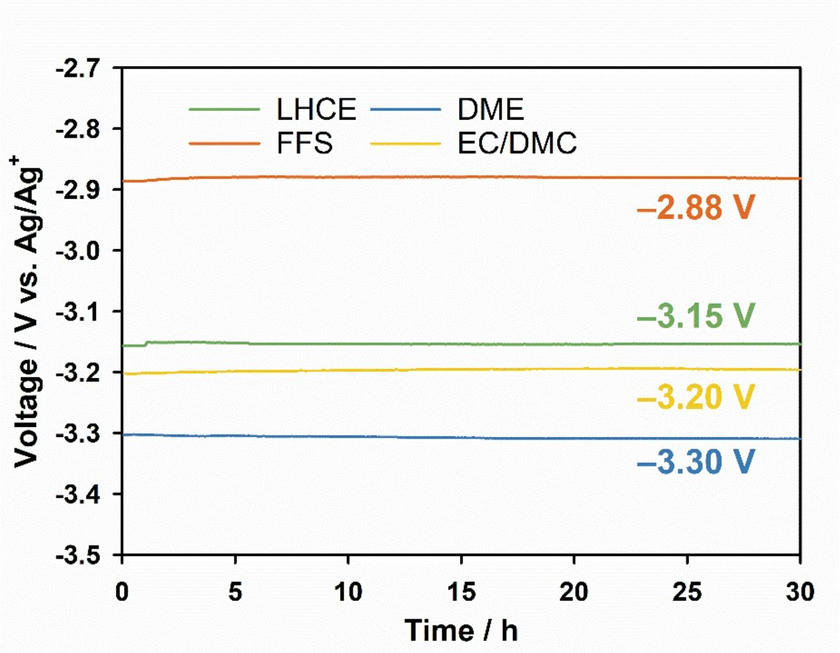

Figure S17. Open-circuit voltage profiles of Li metal electrodes in various electrolytes, such as (i) FFS, (ii) LHCE, (iii) EC/DMC, and (iv) 1 M LiFSI in DME (denoted as DME).
